# A Comparative Study on the Finite Element Analysis of Multilayered Honeycomb Composite Materials for Aerospace Structures

**DOI:** 10.3390/ma18081744

**Published:** 2025-04-10

**Authors:** Miruna Ciolca, Raul Cormos, Catalin Andrei Neagoe, Anton Hadar

**Affiliations:** 1 Department of Strength of Materials, National University of Science and Technology POLITEHNICA Bucharest, Splaiul Independentei 313, 060042 Bucharest, Romania; miruna.ciolca@upb.ro (M.C.); cormosr@yahoo.com (R.C.); 2 INCAS—National Institute for Aerospace Research “Elie Carafoli”, Iuliu Maniu Blvd. 220, 060042 Bucharest, Romania; 3 Department of Dynamic Systems, Institute of Solid Mechanics of the Romanian Academy, Constantin Mille 15, 030167 Bucharest, Romania; 4 Technical Sciences Academy of Romania, Dacia Blvd. 26, 030167 Bucharest, Romania; 5 Academy of Romanian Scientists, Ilfov Street 3, 050045 Bucharest, Romania

**Keywords:** multilayered honeycomb composite material, finite element analysis, aerospace sandwich structures, local analysis, equivalent model

## Abstract

Honeycomb composite materials are widely used in many areas of mechanical engineering where weight saving is a crucial factor. One of the main loads that such material configurations are designed to withstand is compressive load. The most important industrial sector where honeycomb composite materials have found their application is in the aerospace industry, due to their advantages of high strength and lightness. In this article, two forms of finite element model analyses are presented for a novel multilayered honeycomb composite material with impregnated paper cores. The first represents a detailed approach tailored for local analysis, while the second is useful for a global analysis of the honeycomb composite material. Both types of modeling techniques are presented with a description of their advantages and drawbacks, highlighting the increased precision of the complex model—closest deformation estimations—and the agility of the equivalent one—an 80% reduction in complexity, providing acceptable results. An initial comparative analysis is performed, and the obtained results are discussed. An experimental validation is also carried out, followed by the presentation of a suggested practical application, displaying good accordance.

## 1. Introduction

Composite materials have witnessed an important development in the scientific field throughout the 20th century. They are widely used in many areas of mechanical engineering and represent the subject of extensive research. One important type of such material is represented by honeycomb composite materials, which are used in numerous forms [1,2]. These composites are used in various engineering applications due to their superior energy absorption capacity and reduced weight, properties that are very important in aerospace structures [3,4,5,6,7].

A honeycomb sandwich configuration is a structure made up of two relatively thin face sheets attached to a lightweight honeycomb core. The face sheets mainly handle tensile and compressive forces, while the core provides support to prevent the face sheets from buckling and the appearance of out-of-plane shear forces.

Many researchers have analyzed the behavior of the honeycomb sandwich structure using numerical and experimental methods in order to validate the best numerical approach using specialized software that can reduce the processing time of obtaining accurate results [8,9,10,11]. There are engineering simulation software solutions that use the finite element method to test the response of various material configurations under different mechanical loads.

On this topic, Liu et al. investigated the mechanical response of Nomex honeycomb cores under transverse loading, stating that the resin coating volume influences the honeycomb strength at failure, but not its strain [12]. Russell et al. examined deformation and failure modes for honeycombs and observed that, during compression tests, there is a minimal debonding at the interface between the face sheet and the core [13].

Han et al. studied the mechanical behavior under compression and impact loading conditions of CFRP all-composite sandwich structure, using finite element numerical analysis, obtaining comparable results with experimental results from specialty literature [14]. Priyadarsini Morampudi et al.’s review on glass fiber-reinforced polymer composites discussed the properties obtained under mechanical loading for different manufacturing methods. They concluded that the best composite suitable for aerospace applications is glass fiber with epoxy or polyester as matrix material [15].

Pandu et al. took into consideration the experimental analysis of glass fiber-reinforced composite under mechanical loading using FEA software. They used a composite two-wheeler front axle manufactured from glass fiber and epoxy resin materials, subjected to different mechanical loadings, and compared the results with the ones obtained for an axle commonly used in the industry. The tests conducted on the specimen were also analyzed using FEA. As a result, impact and fatigue tests showed better behavior for the composite axle, and also a weight reduction of approximately 60% compared to traditional steel shafts [16].

Because using finite element analysis for complex geometries, such as honeycomb composites, is time-consuming, researchers have tried to reduce computational and time resources with equivalent models. Steenackers and Peeters studied the influence of using an equivalent composite honeycomb model with FEA instead of the classical complex model. They obtained promising results with the equivalent material, with stress values almost identical to the highly detailed model [17].

Honeycomb composite materials are widely utilized in many areas of industry due to their numerous advantages. One of the industries which employs such materials is the aerospace industry, where weight and strength considerations are major factors in the use of composites. These materials can be found in various parts of aircraft components, starting from primary to secondary structural parts, where critical characteristics include light weight, a high strength-to-weight ratio, damping and dimensional accuracy.

Aircraft structures, when flying, are subjected to pressure distributions, which imply the appearance of different loads such as bending, compression, tension, shear or torsion. The usual applications in the aircraft industry for honeycomb composites using paper cores are aircraft flooring loaded in compression due to weight, aircraft interiors (sidewalls, galleys and ceilings), cargo lining subjected to pressure and fuselage components under dynamic compressive forces [18,19,20].

The behavior of honeycomb structures and their potential attachments are a major area of study in both static and dynamic load case scenarios. In this sense, practical and scientific studies were carried out to investigate their performance in a large variety of loading conditions, and great scientific effort is being made in terms of their ongoing research. Herein, a key problem is to predict their behavior using finite element simulations with a minimum of computational effort, when they represent parts of complex structural components [21,22,23,24].

In aerospace applications, one of the main challenges is choosing the right material for the manufacture of the components. The material should have good energy absorption and dissipation properties, relating to the crashworthiness ability of the aerospace structure, which means protecting the passengers and the cargo [25,26,27]. Composite materials are starting to be used more and more often in this industry, being able to respond not only to the energy absorption requirement, but also to low weight, durability and strength criteria. The numerical and experimental tests carried out on honeycomb composite material structures have demonstrated satisfying results that correspond to the strict standards introduced by the aerospace industry [28,29,30].

From the literature review, it can be observed that the majority of studies usually focus on a single numerical model, thus emphasizing the need for comparative studies. Moreover, multilayered sandwich composite materials have scarcely been investigated and simulated. Therefore, the purpose of this article is to present and compare two ways of performing a finite element analysis for a newly developed multilayered honeycomb composite material that can be used in aerospace structures. The advantages and disadvantages of the models are discussed, and the numerical results are analyzed. As a validation procedure, the minimum compressive strain principle [31,32] is used to evaluate the difference between the behavior of the two models.

## 2. Materials and Methods

The first numerical model of the multilayered honeycomb composite material is a full 3D shell representation, formed with a high number of finite elements. The second, less complex model acts as an orthotropic equivalent model of the material, having a single sheet of elements but with all the layers of the first model defined within, in the order of appearance.

The primary approach which presents the modeling of each layer of the composite material is useful for local analysis if, in the structure, a local evaluation is desired. The main disadvantage of this approach is that it is very costly in terms of computational resources.

The advantages of the latter approach are the smaller number of finite elements used and the possibility of studying, in a general sense, the behavior of the composite material. The disadvantage is the fact that the second model does not allow for a potential detailed behavior analysis of the composite material, such as for wall buckling of the honeycomb cores or wrinkling phenomena.

To present the use of the two modeling techniques mentioned, the two finite element models are validated against experimental data. In addition, for both models of the multilayered honeycomb composite material, the benefits and drawbacks are illustrated in a possible practical application.

### 2.1. Material Design and Description

**Figure 1:**
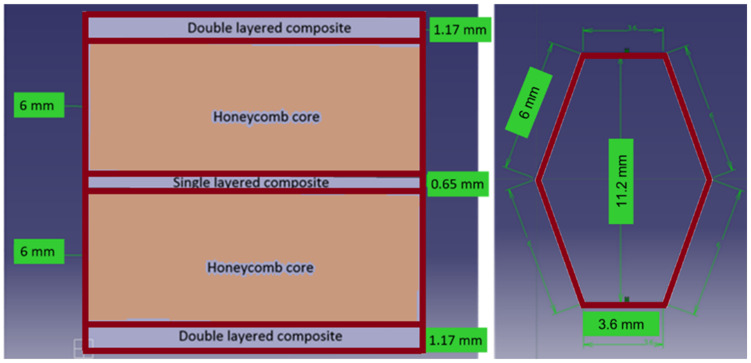
Multilayered honeycomb composite material configuration.

The studied multilayered honeycomb composite was designed and manufactured by the authors at the laboratories of the Department of Strength of Materials. The material specimens have a total thickness of 15 mm and are made out of five layers of materials, as presented in Figure 1.

**Figure 2:**
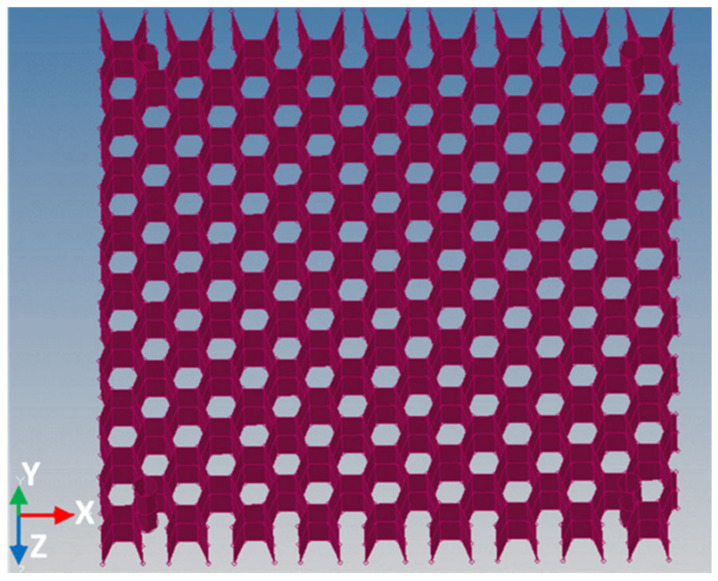
Honeycomb cores arranged in an overlapping position.

The five distinctive layers consist of two double-layered composite sheets made of woven glass fibers impregnated with polyester resin, forming the outer faces, and two honeycomb paper cores impregnated with polyester resin and a 1–1.5% hardener, separated by a single layer of composite sheet, of the same type as the outer double-layered sheets. The joining between the components was made with the same type of polyester resin. The honeycomb core has a hexagonal shape and a wall thickness of 0.23 mm. The two honeycomb cores are situated in an overlapping position, as shown in Figure 2, to increase the bending and compressive stiffness.

The material characteristics of the outer and middle glass fiber composite sheet layers were determined previously by the authors in experimental tests [33,34] and are reported here in Table 1.

For the impregnated honeycomb paper core, the isotropic material characteristics are presented in Table 2 [33]. The elastic modulus was determined experimentally by performing tensile tests on several core specimens. Compared to an unimpregnated paper core, the hardened paper displayed a stiffer and more linear response.

### 2.2. Computation of Equivalent Orthotropic Material Data for the Honeycomb Core

Considering that the honeycomb core has a spatial 3D configuration and cannot be used as such in a reduced model, it is necessary to compute the equivalent orthotropic data for the 2D model. Thus, the mathematical model described by Gibson and others is applied [35,36,37,38]. The starting point is the core material data, considered isotropic, as well as the cell geometric data.

The mechanical material data are computed, as shown in the following formulae:

1. Transverse elastic modulus for the cell wall:

(1) $$G=\frac{E}{2\left(1+\upsilon \right)}$$

where:

E—longitudinal elastic modulus of the material;
ν—Poisson’s ratio.

**Figure 3:**
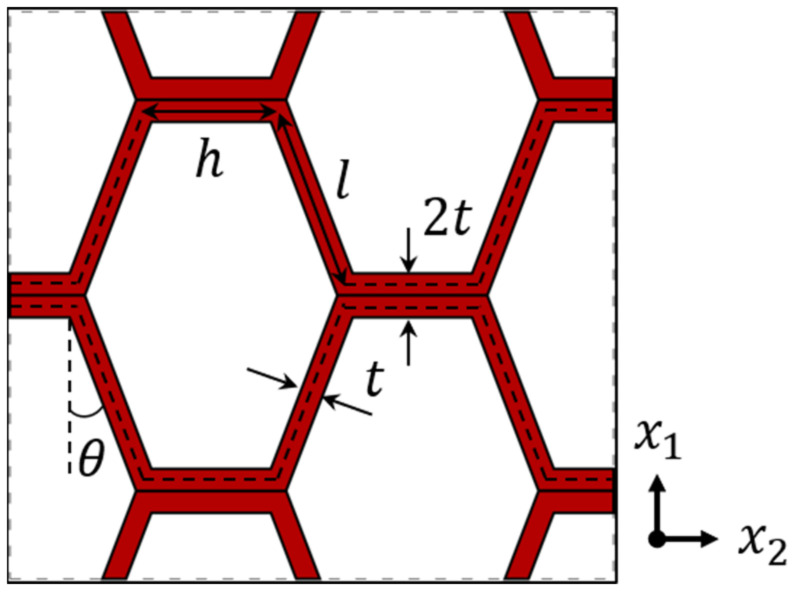
Geometric parameters of honeycomb cell walls.

The explicit geometric parameters of the honeycomb are presented in Figure 3.

2. Modulus of elasticity of orthotropic honeycomb core in x_1_ direction:
  (2) $$E_{1}^{\prime }=E{\left(\frac{t}{l}\right)}^{3}\frac{\cos\left(\theta \right)}{\left(\frac{h}{l}+\sin\left(\theta \right)\right)\sin^{2}\left(\theta \right)}$$
3. Modulus of elasticity of orthotropic honeycomb core in x_2_ direction:
  (3) $$E_{2}^{\prime }=E{\left(\frac{t}{l}\right)}^{3}\frac{\left(\frac{h}{l}+\sin\left(\theta \right)\right)}{\cos^{3}\left(\theta \right)}$$
4. In-plane Poisson’s ratio:
  (4) $$\upsilon_{12}^{\prime }={\left(\upsilon_{21}^{\prime }\right)}^{-1}=\frac{\cos^{2}\left(\theta \right)}{\left(\frac{h}{l}+\sin\left(\theta \right)\right)\sin\left(\theta \right)}$$
5. Transverse shear modulus on x_1_-x_2_ plane:
  (5) $$G_{12}^{\prime }=E{\left(\frac{t}{l}\right)}^{3}\frac{\left(\frac{h}{l}+\sin\left(\theta \right)\right)}{{\left(\frac{h}{l}\right)}^{3}\left(1+\frac{h}{4l}\right)\cos\left(\theta \right)}$$
6. Elastic modulus in out-of-plane direction:
  (6) $$E_{3}^{\prime }=E\left(\frac{t}{l}\right)\frac{\left(\frac{h}{l}+1\right)}{\cos\left(\theta \right)\left(\frac{h}{l}+\sin\left(\theta \right)\right)}$$
7. Out-of-plane Poisson’s ratios:
  (7) $$\upsilon_{23}^{\prime }=\upsilon_{13}^{\prime }=\upsilon$$
8. x_1_-x_3_ plane shear modulus:
  (8) $$G_{13}^{\prime }=G\left(\frac{t}{l}\right)\frac{cos\left(\theta \right)}{\left(\frac{h}{l}+\sin^{2}\left(\theta \right)\right)}$$
9. x_2_-x_3_ plane shear modulus:
  (9) $$G_{23}^{inf}\geq G\left(\frac{t}{l}\right)\left(\frac{\frac{h}{l}+\sin\left(\theta \right)}{\left(\frac{h}{l}+1\right)cos\left(\theta \right)}\right)$$
  (10) $$G_{23}^{sup}\leq G\left(\frac{t}{l}\right)\left(\frac{\frac{h}{l}+\sin^{2}\left(\theta \right)}{\left(\frac{h}{l}+sin\left(\theta \right)\right)cos\left(\theta \right)}\right)$$
  (11) $$G_{23}^{\prime }=G_{23}^{inf}+\frac{0.787}{\frac{h}{l}}\left(G_{23}^{sup}-G_{23}^{inf}\right)$$

Taking into account that the θ wall angle was measured as equal to 20°, the results included in Table 3 were calculated and entered into the second finite element model for the equivalent orthotropic material data of the honeycomb cores.

For the first finite element model, which relies on the 3D shell representation of the cores, the data shown before in Table 2 were introduced for the honeycomb cores, considering the impregnated paper material to be isotropic.

### 2.3. Experimental Investigation of the Honeycomb Structure

The finite element model results, which will be discussed further on, were compared using experimental tests on composite materials, which present the behavior of the material under quasi-static concentrated compressive loading. These tests are meant to validate the behavior displayed by the finite element models on the linear elastic domain of the material. This validation allows for a proper future use of the finite element models for multilayered honeycomb composite materials in practical applications.

Quasi-static local compressive tests can be considered, as well, to better understand the potential energy absorption capacity of the novel multilayered composite material under a low-velocity impact, as previously researched and reported by the authors in [39].

**Figure 4:**
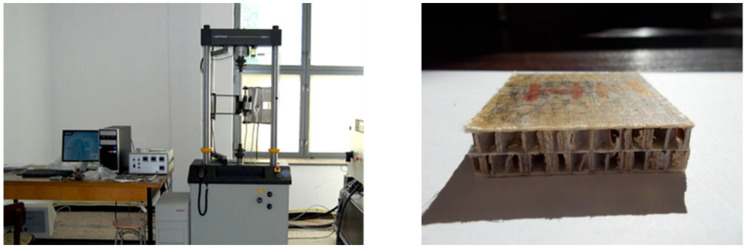
Testing machine used for experiments (**left**) and a multilayered honeycomb composite specimen (**right**).

To evaluate the behavior of the multilayered honeycomb structure, an experimental determination was made to obtain the material force–displacement curve. For the experimental tests, an Instron 8800 testing machine was used, capable of producing 100 kN of force. To apply the point load, a purposefully made conic testing head was used, with a head radius of 10 mm. Figure 4 shows the testing machine and one of the specimens before completing the experiments.

The tests were performed on three specimens, under standard conditioning and testing laboratory conditions, according to ISO 291:2008 [40]. Each sample was set on a metallic support, and a slow displacement of 1 mm/min of the head was introduced, recording the force versus displacement curve. The specimen’s edges were fixed during the loading phase, and the displacement was measured at the center of the plate, where the load was applied.

**Figure 5:**
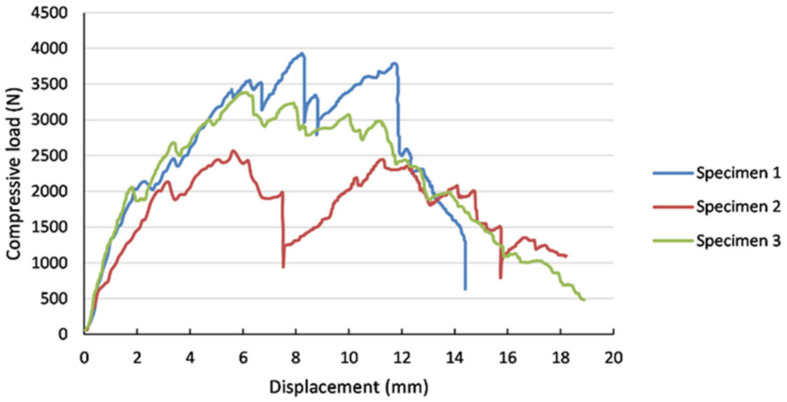
Experimental force–displacement curves for the multilayered honeycomb composite material.

The force–displacement curves obtained for the three specimens after performing the tests are presented in Figure 5.

After analyzing the test results, it is possible to observe that the linear elastic domain is within the range of 0 to about 2000 N. Specimen no. 2 presented an earlier onset of failure due to possible unevenness of the applied hardening agent.

### 2.4. Finite Element Models of the Experimental Setup

To study the behavior of the multilayered honeycomb structure, two detailed finite element models were created. This approach was used to improve result accuracy and the convergence of the analysis.

The two finite element models were made using the Altair HyperMesh 2021 preprocessor in conjunction with the Nastran 2023 solver [41,42,43]. Patran 2023 software was used for post-processing [44].

**Figure 6:**
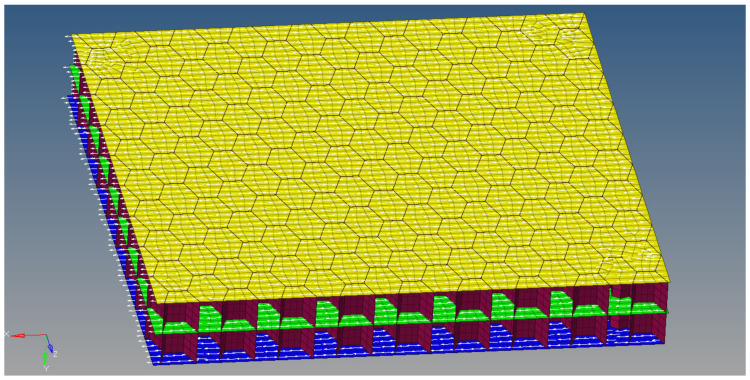
First finite element model for the multilayered honeycomb composite specimen.

The first finite element model of the multilayered honeycomb structure is made of shell elements, with the laminate composite orientation along the global X axis direction. The element size is 0.5 mm with a total of 2,460,016 elements. The specimen’s dimensions are the same as those used in the experimental test, 60 mm × 60 mm. The joining between the core and the sheets is assured considering a node-on-node connection. Figure 6 shows the first finite element model developed for the multilayered honeycomb composite material.

**Figure 7:**
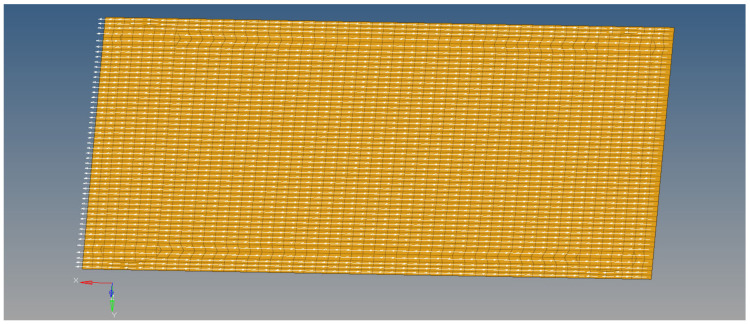
Second, equivalent multilayer finite element model, defined on a single sheet of elements.

The second model is a single-sheet model that has all five layers of composite plies defined, with the orientation of the material in the global X axis direction. The element size is 0.12 mm, with a total of 474,112 elements. The element normal axis was considered in the global Y axis direction. Figure 7 shows the single-sheet model for the multilayered material.

**Figure 8:**
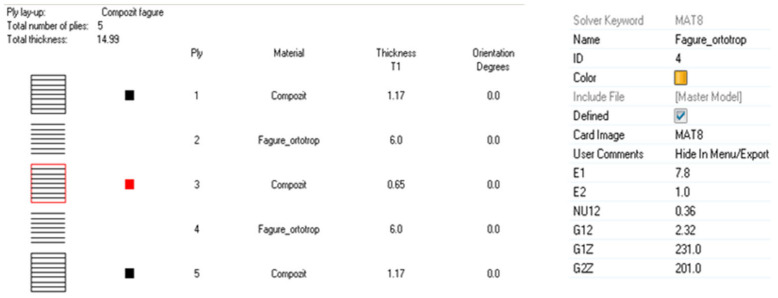
Nastran orthotropic card data input for the multilayered single-sheet composite.

The honeycomb material and layer properties for the second model are presented in Figure 8. The connection between the equivalent honeycomb layers is made by node-on-node correspondence.

### 2.5. Loading Conditions

**Figure 9:**
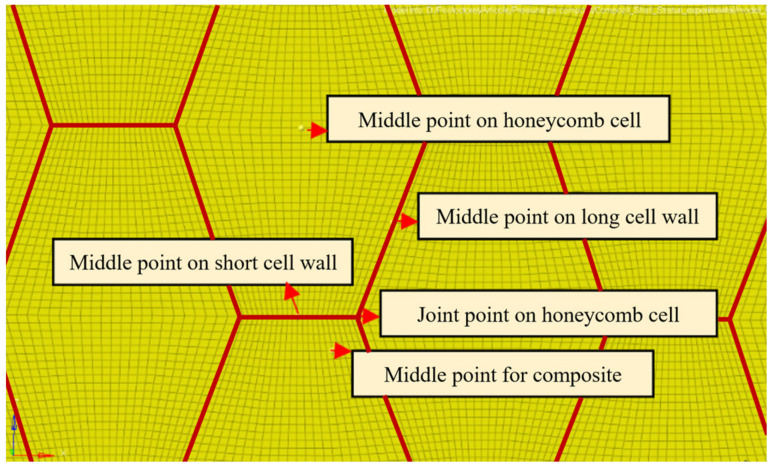
Loading conditions for the multilayered shell-modeled honeycomb composite.

For both models, the outer node edges are blocked in all three translational directions, and a 1000 N load is applied. For the multilayered 3D shell composite, the lower composite layer has the outer edge nodes blocked, and five load cases are considered for the applied load, depending on the load head position relative to the honeycomb core. The five loading points were considered to evaluate the influence of honeycomb stiffness. Figure 9 shows the loading conditions for the multilayer specimen.

### 2.6. Numerical Results

From the experimentally registered curves, it can be observed that, in the linear elastic domain, for a 1000 N load, an average central displacement of approximately 0.5 mm is expected.

**Figure 10:**
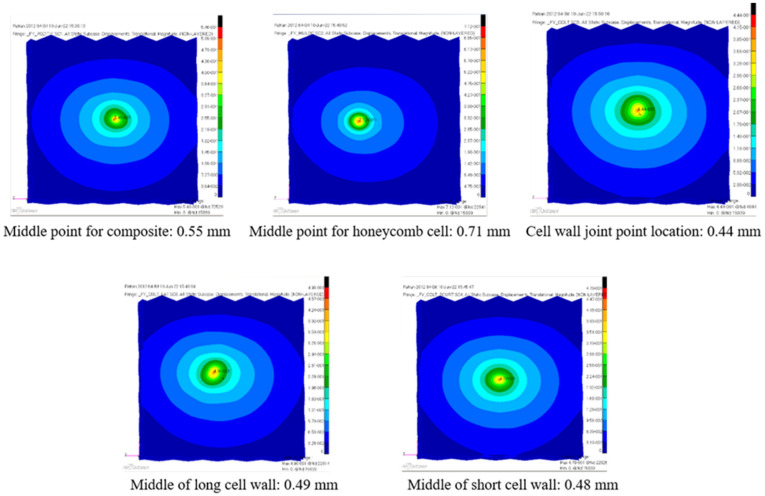
Maximum displacement results for the multilayered finite element model.

For the purposes of comparison, for the multilayered composite model, the numerical results are shown in Figure 10.

**Figure 11:**
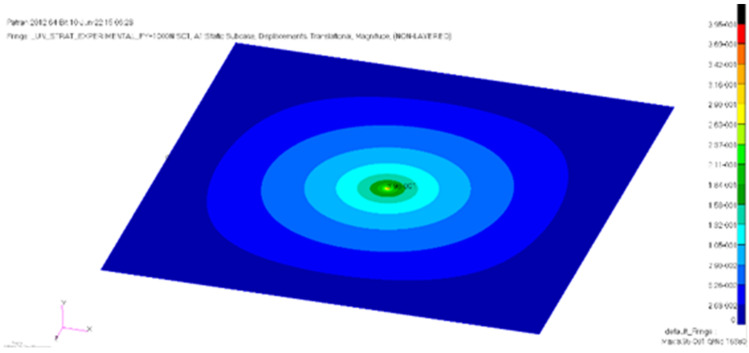
Maximum central displacement for the single-sheet finite element model: 0.40 mm.

The estimated central displacement for the first model is 0.55 mm. In contrast, for the single-sheet finite element model, a 0.40 mm displacement was calculated. Figure 11 shows the finite element simulation for the single-sheet model, under the concentrated local load.

**Figure 12:**
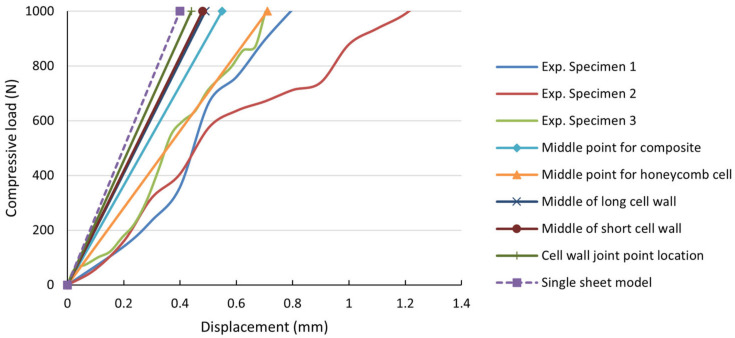
Comparison between the finite element simulation results of the two proposed models and the experimental results.

In Figure 12, the numerical results computed using the two conceived finite element models are compared with the average experimental results obtained from the laboratory tests. The load–displacement data obtained from varying the load position, as illustrated in Figure 5, are also plotted. As the focus of the models is within the linear elastic domain, a maximum load of 1000 N was chosen in the analysis.

Under certain conditions, the finite element model results demonstrate good correlation with the experimental data, indicating that complex models can capture the behavior of the multilayered honeycomb composite material subjected to local compressive loads. In this sense, the closest match was obtained from the first model—with deltas less than 10.9%, considering the point load placed on the middle of the honeycomb cell, while the other finite element curves, including that of the single-sheet model, illustrate a stiffer virtual behavior.

## 3. A Practical Case Analysis for Multilayered Honeycomb Composite Materials

In practical applications, when parts of complex structures are made from composite materials, the multilayered or the detailed FEM approaches are not used due to the increased model size. Usually, the single-sheet definition of the composite layers is used in a global finite element model approach, with a coarser mesh size. In addition, pressure loads are also used more commonly than point loads.

In this section, the following analysis of the multilayered honeycomb composite material presents how the composite models are simulated in real structures, and afterward the results are compared with the multilayered simulations. The practical application may be interpreted as representing the following cases:

- static loading given by a weight, for example, in aircraft floor loading;
- dynamic pressure acting on structure, given by the vicinity of a moving fluid or aerodynamic forces;
- static pressure given by a stationary fluid, such as tank pressure loads.

### 3.1. Finite Element Model Description

**Figure 13:**
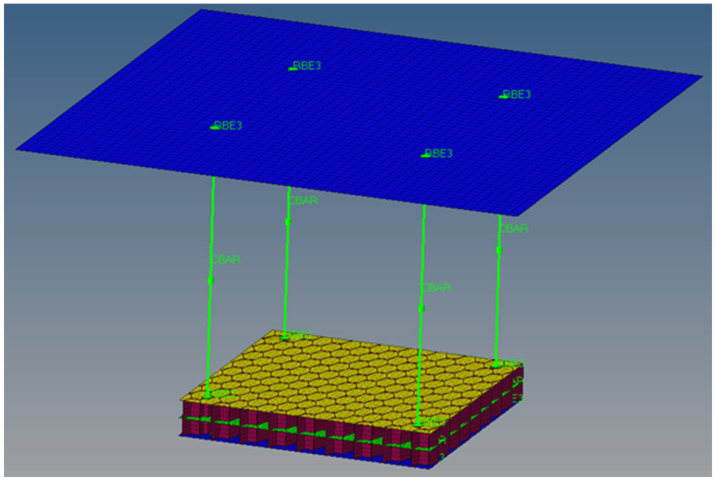
Detailed finite element model of the multilayered honeycomb composite material.

Figure 13 shows the finite element model of the multilayered honeycomb composite material.

The model consists of a multilayered composite material piece that is fastened at the corners to a top steel plate. On the upper steel plate, which has standard steel material properties and a thickness of 80 mm, a single point constraint (SPC) was defined with all degrees of freedom blocked. The joining between the plate and the finite element model of the multilayered honeycomb composite material is achieved using four CBar elements with a diameter of 4 mm, with RBE3 elements blocked on translation. The honeycomb cores are modeled with four elements in height, for each of the two cores. The orientation for the laminated composites is at 0° in the in-plane X axis direction of the global coordinate system. Thus, the angle was defined on the θ angle for the CQuad card.

The plane size of the composite is 100 mm × 100 mm. The total number of elements is 19,747. The connection with the RBE3s is made only on the upper and lower plate.

**Figure 14:**
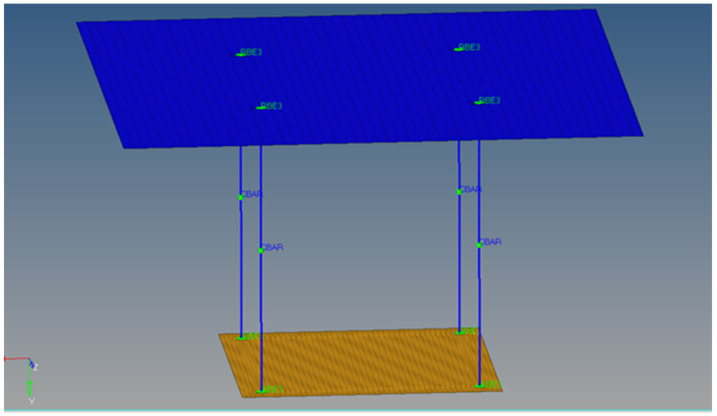
Multilayered honeycomb composite material, defined on a single sheet of elements.

The second model is made of a single layer of elements, where the composite material orientation is defined in the X axis direction, and the layers’ orientation is defined in the element’s normal direction, the Y axis. The total number of elements for this configuration is 2417. The simplified finite element model is shown in Figure 14.

### 3.2. Loading Conditions

To compare the behavior of the two multilayer honeycomb composite structures, the following loading conditions were defined. An SPC on all components on the outer part of the support part was defined. A pressure load card is used to introduce a pressure load of 0.5 MPa that corresponds to a 5000 N load. The load was introduced on the first layer.

### 3.3. Simulation Results

Considering that the honeycomb plate has dimensions of 100 mm × 100 mm, a 60 mm × 60 mm area was chosen in the middle part of the composite. This area was established for result recovery and to avoid singularity results at the composite’s edges, due to the many triangular elements and less regular mesh of the model.

**Figure 15:**
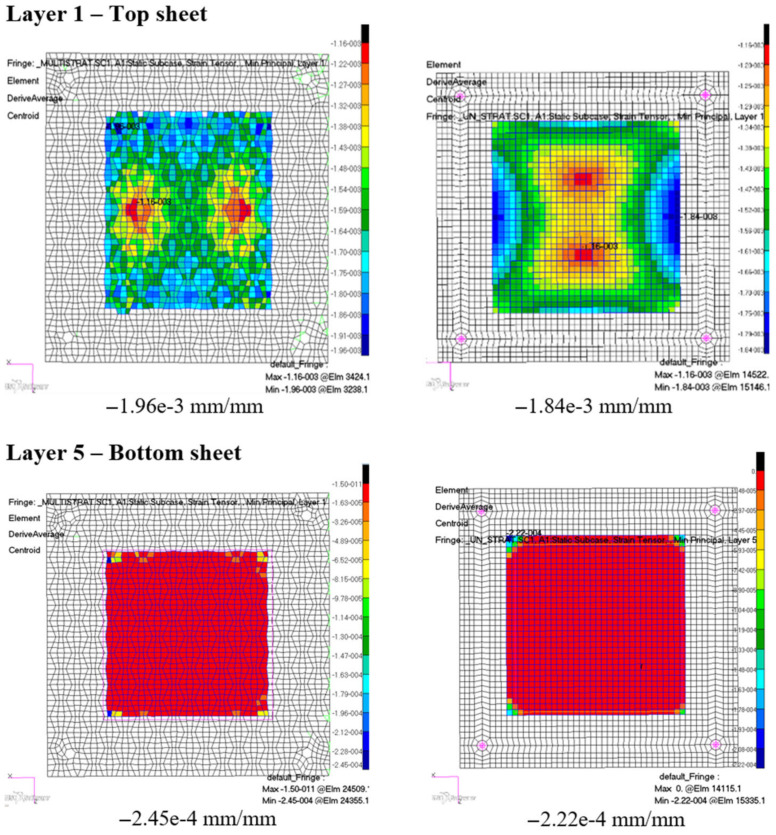
Maximum compressive strains for the top and bottom layers of the composite material: the results computed with the multilayered FE model are on the left, while the results computed with the equivalent single-sheet FE model are on the right, for each layer.

**Figure 16:**
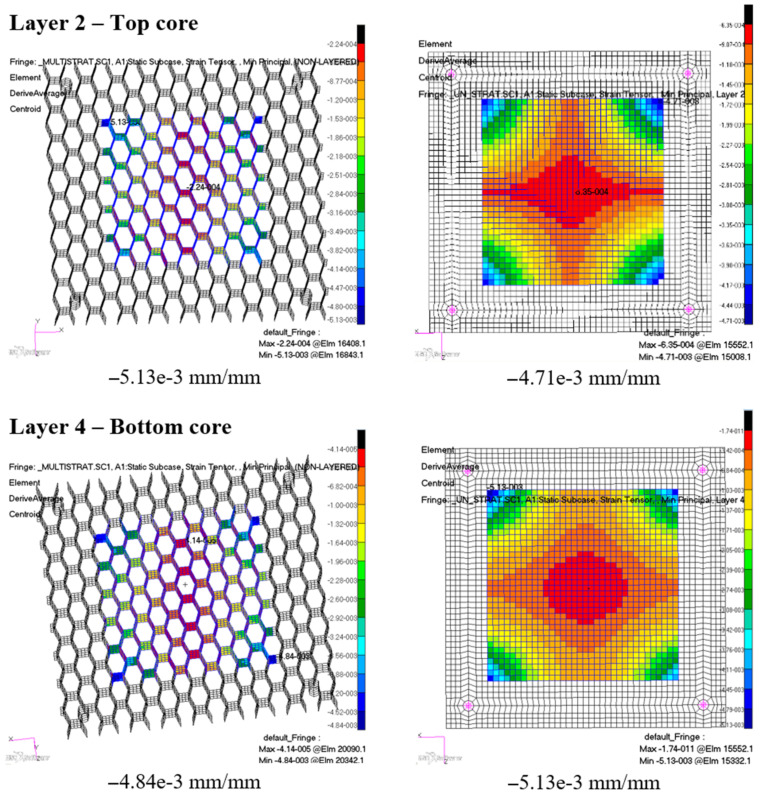
Maximum compressive strains for the top and bottom cores of the composite material: the results computed with the multilayered FE model are on the left, while the results computed with the equivalent single-sheet FE model are on the right, for each layer.

A comparison was made between the two finite element models considering the minimum strain criteria, which is the maximum compressive strain value. The strain distributions are plotted in the following comparative images, in Figure 15 and Figure 16, for each of the distinct material layers of the honeycomb composite material. In each figure, the left image represents the multilayered composite, and the right image the single-sheet composite.

The numerical models predict close maximum compressive strains for the different material layers of the honeycomb composite, with a difference of up to 9.3%. The second model, which has a more simplified approach, has a tendency to offer lower maximum strain results.

## 4. Conclusions

The current article has presented and evaluated two distinct methods for carrying out a finite element analysis to study the behavior of a novel multilayered honeycomb composite material subjected to out-of-plane concentrated compressive loads. The first method consisted of a large FE model with a detailed representation of the composite material cores, while the second method employed a simplified model, with a reduced geometry and equivalent orthotropic material properties.

By analyzing all the obtained results, the following conclusions can be drawn:

- the two FE models offer comparable results when it comes to predicting the compressive behavior under quasi-static loads, each of them presenting different advantages and disadvantages in terms of execution and precision;
- a more detailed analysis, such as the one performed on the first model, can offer better results with reference to local stiffness evaluation or stability phenomena—with differences of less than 11% compared to experimental deflection data. On the other hand, this method is less practical for large structural applications since the modelling complexity is increased fivefold over the equivalent one;
- in contrast, the simplified model, as seen from the results, is better suited for modelling complex assemblies or parts, where a global behavior analysis is more desired. This method relies on an appropriate estimation of the equivalent mechanical orthotropic properties for the composite material and can offer results close to those of the detailed model, up to 9% difference, with a reduction of up to 80% of computational time. While this second model can give reliable information about structural integrity, it cannot truly predict local effects;
- numerical results were validated successfully with experimental data obtained from laboratory tests, displaying a suitable accuracy for the linear elastic domain, before the material’s staggered failure. A comparison between the two previous modelling techniques was also made considering the minimum compressive principal strain that was registered, and a good correlation was found for the two approaches.

The study has also highlighted the need for further numerical comparative investigations regarding local instability phenomena and nonlinear material behavior.

## Figures and Tables

**Table 1:** Glass fiber sheet material data.

Characteristics	Value	Unit of Measurement
$E_{x}$	16,954	[MPa]
$E_{y}$	14,684	[MPa]
$E_{z}$	7122.9	[MPa]
$\upsilon_{xy}$	0.129	
$\upsilon_{zy}$	0.109	
$\upsilon_{zx}$	0.33	
$G_{xy}$	5942.3	[MPa]
$G_{yz}$	5014.3	[MPa]
$G_{zx}$	3138.5	[MPa]

**Table 2:** Mechanical characteristics of the impregnated honeycomb core wall.

Characteristics	Value	Unit of Measurement
E	16,357	[MPa]
G	6058	[MPa]
ν	0.35	

**Table 3:** Equivalent orthotropic material data for the honeycomb cores.

$E_{1}^{\prime }$ [MPa]	$E_{2}^{\prime }$ [MPa]	$E_{3}^{\prime }$ [MPa]	$\upsilon_{12}^{\prime }$	$\upsilon_{21}^{\prime }$	$\upsilon_{23}^{\prime }$ $=\upsilon_{13}^{\prime }$	$G_{12}^{\prime }$ [MPa]	$G_{13}^{\prime }$ [MPa]	$G_{23}^{\prime }\;[\mathrm{MPa}]$
7.86	1.05	1133.32	2.74	0.36	0.35	2.32	231.66	201.37

## Data Availability

Dataset available on request from the authors.
